# Results of a randomized, double blind, placebo controlled, crossover trial of melatonin for treatment of Nocturia in adults with multiple sclerosis (MeNiMS)

**DOI:** 10.1186/s12883-018-1114-4

**Published:** 2018-08-06

**Authors:** Marcus J. Drake, Luke Canham, Nikki Cotterill, Debbie Delgado, Jenny Homewood, Kirsty Inglis, Lyndsey Johnson, Mary C. Kisanga, Denise Owen, Paul White, David Cottrell

**Affiliations:** 1 School of Clinical Sciences, University of Bristol, Bristol, UK; 2 Bristol Urological Institute, Southmead Hospital, Bristol, BS10 5NB UK; 3 Neurology Department, Southmead Hospital, Bristol, BS10 5NB UK; 4 University of the West of England, Bristol, UK

**Keywords:** Nocturia, Multiple sclerosis, Melatonin, Antidiuretic, Antimuscarinic, LUTS

## Abstract

**Background:**

Nocturia is a common urinary symptom of multiple sclerosis (MS) which can affect quality of life (QoL) adversely. Melatonin is a hormone known to regulate circadian rhythm and reduce smooth muscle activity such as in the bladder. There is limited evidence supporting use of melatonin to alleviate urinary frequency at night in the treatment of nocturia. The aim of this study was to evaluate the effect of melatonin on the mean number of nocturia episodes per night in patients with MS.

**Methods:**

A randomized, double blind, placebo controlled crossover trial was conducted. 34 patients with nocturia secondary to multiple sclerosis underwent a 4-day pre-treatment monitoring phase. The patients were randomized to receive either 2 mg per night (taken at bedtime) of capsulated sustained-release melatonin (Circadin®) or 1 placebo capsule for 6 weeks followed by a crossover to the other regimen for an additional 6 weeks after a 1-month washout period.

**Results:**

From the 26 patients who completed the study, there was no significant difference observed in the signs or symptoms of nocturia when taking 2 mg melatonin compared to placebo. The primary outcome measure, mean number of nocturia episodes on bladder diaries, was 1.8/night at baseline, and 1.4/night on melatonin, compared with 1.6 for placebo (Medians 1.70, 1.50, and 1.30 respectively, *p* = 0.85). There was also no significant difference seen in LUTS, QoL and sleep quality when taking melatonin. No significant safety concerns arose.

**Conclusions:**

This small study suggests that a low dose of melatonin taken at bedtime may be ineffective therapy for nocturia in MS.

**Trial registration:**

(EudraCT reference) 2012–00418321 registered: 25/01/13. ISRCTN Registry: ISRCTN38687869.

## Background

Nocturia is the complaint that the individual has to wake at night one or more times to void [1]. It can result from a range of factors, including behavioural influences, sleep disturbances, lower urinary tract dysfunction and altered fluid or salt homeostasis. Nocturia is prevalent in the general population and is known to increase in severity with age. 77% of people aged 60 and above experience some degree of nocturia with no difference seen between men and women [2]. Nocturia impacts greatly on quality of life (QoL), potentially due to fatigue, cognitive dysfunction and disturbed emotional health [3]. Furthermore, severe nocturia may be associated with cardiovascular disease, autonomic disease, obstructive sleep apnoea and chronic kidney disease [4], and potentially a higher risk of mortality [5]. A very high proportion of MS patients have lower urinary tract symptoms (LUTS) [6]. LUTS are a substantial problem in MS, and nocturia is a particularly prominent symptom with substantial detrimental impact on QoL [7].

Current treatments for nocturia include managing fluid intake, timed diuretics, desmopressin, antimuscarinic drugs, bedtime sedatives and miscellaneous compounds [8]. Desmopressin is indicated for treatment of nocturia in MS [9]. However, desmopressin can cause hyponatraemia [10] and has been associated with hyponatraemic convulsions [11, 12]. Indeed, it is recommended that tri-cyclic antidepressants (commonly used in MS patients) are avoided when using Desmopressin to reduce the risk of hyponatraemia (British National Formulary). This can also potentially be an issue with diuretics. Antimuscarinics are known to cause dry mouth, constipation, swallowing difficulty and confusion. Patients taking sedatives can experience hangover sedation, while elderly subjects are at risk of cognitive impairment [13]. These side effects and poor efficacy mean that clinicians are sometimes reluctant to initiate treatment for nocturia.

Melatonin (N-acetyl-5-methoxytryptamine) is a hormone secreted primarily at night by the pineal gland. It regulates circadian rhythms and reduces smooth muscle spontaneous activity, including that found in the bladder [14]. Melatonin tablets taken before bedtime may reduce nocturia in a subgroup of patients with benign prostate enlargement [15]. In elderly patients with nocturia, levels of severity and QoL improve with melatonin use [16]. In MS, sleep quality is commonly reduced as a consequence of a wide range of sleep abnormalities, of which nocturia is only one example. In MS there can be an impairment of endogenous melatonin secretion [17, 18], and administration of oral melatonin improves reduced sleep quality in MS patients [19].

We hypothesised that bedtime administration of a melatonin sustained-release tablet will improve clinical nocturia in patients with MS. We previously published the protocol of the “Melatonin for nocturia in MS (MeNiMS)” study to evaluate this hypothesis [20], and the current study reports the findings. We chose a low dose of 2 mg, as melatonin levels negatively correlate with multiple sclerosis activity in humans, and alterations in endogenous melatonin have been proposed potentially to affect MS relapses [21]. The primary aim was to evaluate the effect of melatonin on mean number of nocturia episodes per night in MS patients. The secondary aims included: 1) improvement in QoL, 2) safety, 3) LUTS, 4) sleep quality and 5) total voided (urinated) volume and mean volume per void. A qualitative study was also included, which will be reported separately.

## Methods

The detailed study protocol has previously been published [20]. In brief, male and female patients aged ≥18 were recruited at Southmead Hospital, Bristol, UK. Each patient had a confirmed neurological diagnosis of MS as per the 2010 McDonald MS Criteria [22]. They also reported at least one episode of nocturia every night on the International Consultation on Incontinence Questionnaires (ICIQ) Nocturia questionnaire (ICIQ-N [23]). Patients were excluded if they had (i) an indwelling urinary catheter; (ii) used desmopressin or investigational medications in the month preceding randomization; (iii) taken antimuscarinic or diuretic medication, unless used long-term prior to study (> 3 months) and continued throughout the study; (iv) taken melatonin on prescription or purchased; (v) used “sleeping tablets” on prescription or purchased; (vi) diabetes mellitus/diabetes insipidus; and (vii) or if they were pregnant at screening, or of child-bearing potential and unwilling to use contraception. Dipstick urinalysis to exclude urinary tract infection was undertaken at screening. The U.K. National Research Ethics Service Committee South West – Exeter approved the study protocol (REC reference number: 12/SW/0322).

**Fig. 1:**
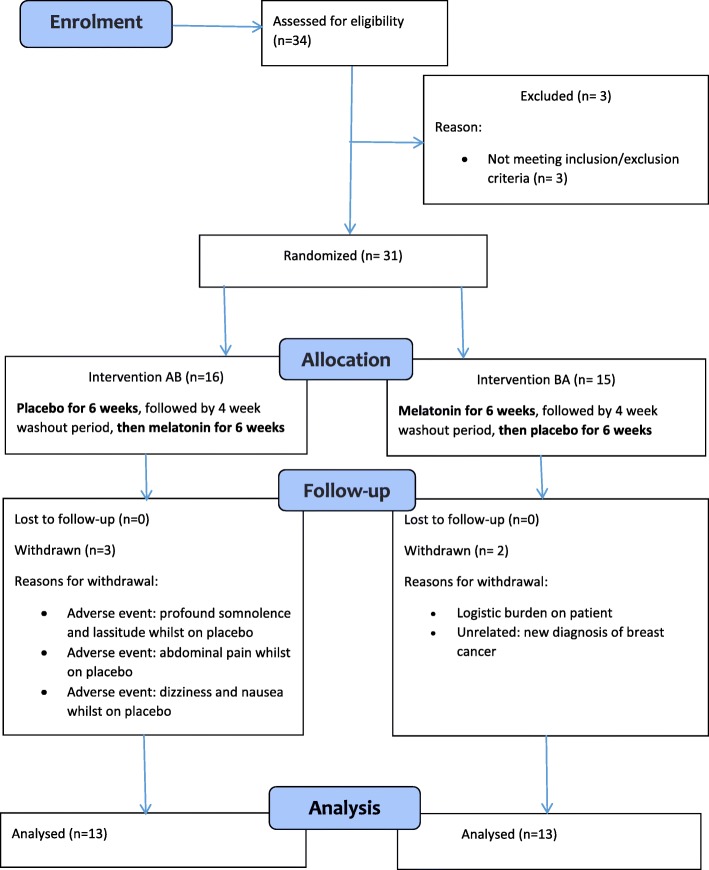
Study flow chart

This was a randomized, double blind, placebo controlled crossover trial with two groups (Fig. 1). Following an initial four-day pre-treatment monitoring phase, the treatment phases were 2 mg per night of capsulated sustained-release melatonin (‘Circadin’) or an identical placebo capsule per night for 6 weeks each, separated by a 4 week washout period. Patients were allocated double-blind via a website (http://www.randomization.com) to group AB or BA, with unblinding undertaken following database lock and analysis (A = placebo, B = melatonin).

The primary outcome was reduction in nocturia episodes per night, derived from the ICIQ bladder diary (ICIQ-BD) [24]. Secondary outcomes included; 1. Subjective severity, using the ICIQ tools on nocturia (ICIQ-N) and nocturia quality of life (ICIQ-NQoL) [25]. 2. MS quality of life, assessed with the MSQoL scale. 3. LUTS, assessed with the ICIQ-MLUTS and ICIQ-FLUTS for males and females respectively. 4. Sleep quality; measured with the Pittsburgh Sleep Quality Index (PSQI). 5. Safety, based on adverse event reporting and Expanded Disability Status Scale (EDSS) score [26]. Outcome measures were completed at baseline and at the end of each treatment phase. Adverse event reporting was undertaken throughout, and followed up until resolution or for 3 months [20].

### Statistical analysis

We calculated that for a two-sided test, using standard levels of statistical significance (alpha = 0.05), a sample size of *n* = 21 complete data sets would have 80% power to detect a medium to large effect size (Cohen’s d = 0.65) with 80% power, and a sample size of *n* = 34 would be needed for a medium effect size (Cohen’s d = 0.5) [20].

The balanced two group, two period, two sequence, double-blind, randomised crossover design with wash-out period comparing treatment to control, ranks highly in the hierarchy of designs. The analyses of the resultant data under this AB/BA design may proceed using nonparametric (Mann-Whitney-Wilcoxon *U*) two-sample statistical techniques which assess for carryover effects, period effects, and treatment effect accounting for any period effects using independent samples designs [27] or using paired samples in the absence of period and carryover effects. Treatment effect sizes have been quantified and converted to Cohen’s *d*. For Cohen’s standardized statistic, *d* = 0 indicates the absence of an effect. For statistically significant effects, some broad and cautious threshold guidance to aid interpretation is for, 0 < *d* < 0.1 to indicate a trivial effect, 0.1 < *d* < 0.3 to indicate a small effect, 0.3 < *d* < 0.5 to indicate a moderate effect, 0.5 < *d* < 0.8 a medium size effect, 0.8 < *d* < 1.3 a large effect, and *d* > 1.3 a very large effect [28]. A missing data analysis was also performed on the primary outcome measure to assess sensitivity of statistical conclusions to missingness. This analysis indicated any missing data to be consistent with being missing completely at random (MCAR). Multiple Imputation by Chained Equations (MICE) [29] with 1000 imputed data sets was performed. These imputed analyses faithfully reproduced the findings from the observed sample data and for brevity of exposition, and to avoid redundancy, are not reported in full.

## Results

In total 13 men and 18 women of mean age 54.8 years (range 34–69) were randomised. Five patients had Primary Progressive MS, 15 patients had Secondary Progressive MS and 11 patients had Relapsing Remitting MS (RRMS). Five of the 11 with RRMS were taking disease modifying therapy at the time of the trial (one interferon beta-1a, two fingolomid and two dimethyl fumarate). Mean EDSS was 4.2 (median 4.0, range 1.5 to 8.0). Five patients withdrew from the trial (Fig. 1). Reasons for withdrawal were; adverse events (three patients), new-onset unrelated health problems (one patient) and logistic burden (one patient). Mean nocturia severity at baseline from the bladder diary was 1.78 episodes/night and self-reported mean ICIQ symptom score for nocturia was 1.80 episodes/night (range 1–3).

The effects of 2 mg melatonin and placebo on bladder diary parameters are shown in Table 1. There was no significant change seen with melatonin for the primary outcome measure, the number of nocturnal episodes per night. “Objective” nocturia was 1.4/ night for melatonin, compared with 1.6 for placebo (U = 43, *p* = 0.85). Average nocturnal output and nocturnal polyuria index (NPI) were also not significantly altered. Effects on patient-reported LUTS are shown in Table 2. For patient-reported (subjective) nocturia, the number of episodes per night after 6 weeks of melatonin was 3.3, compared with 3.2 with placebo. Overall scores and individual LUTS were not significantly different with melatonin compared with placebo.

**Table 1:** Median and range for average number of nocturia episodes, voided output, and NPI by treatment with treatment *p*-value and standardized effect size, *d*

Measure	Treatment							
	Baseline		A: Placebo		B: Melatonin		*p*	*d*
	Median	Range	Median	Range	Median	Range		
Average Nocturia Episodes	1.70	1–3	1.50	0–3	1.30	0–3.3	0.618	0.136
Average nocturnal urine output (mls)	741	310–1416	651	300–1933	667	200–1100	0.939	0.020
24-h voided volume (mls)	2125	1200–4000	2213	1250–3900	2000	881–3600	0.254	0.314
Nocturnal Polyuria Index NPI	0.32	0.15–0.71	0.33	0.17–0.56	0.32	0.15–0.60	0.849	0.052

**Table 2:** Median and range of the grouped sub-scores (Voiding and Incontinence) and the individual symptom scores for nocturia, urgency and frequency from the ICIQ LUTS questionnaires (MLUTS and FLUTS for males and females respectively) by treatment, with *p*-values and standardized effect size, *d*

Measure	Treatment					
	A: Placebo		B: Melatonin		*p*	*d*
	Median	Range	Median	Range		
Voiding sub-score	4.0	0–12	2.0	0–12	0.096	0.492
Incontinence (Storage) sub-score	3.0	0–9	3.0	0–12	0.107	0.450
Nocturia	2.0	1–3	2.0	1–3	0.892	0.037
Urgency	2.0	0–3	2.0	1–4	0.772	0.079

Secondary end points looking at QoL also revealed that melatonin did not significantly affect outcomes. The ICIQ-NQoL questionnaire mean totals were 22.1 for melatonin, compared with 23.6 for placebo (*U* = 41.5, *p* = 0.34; Table 3). For the MSQoL scale (Table 4), there was no significant change between melatonin and placebo in most of the domains, except for physical overall score (50.9 vs. 47.9, respectively, median 48.5 vs 44.0, *U* = 26, *p* = 0.02), role limitations due to physical problems (39.1 vs. 33.3, median 25 vs 12.5, *U* = 29, p = 0.02) and pain score (62.7 vs. 70.6, median 70 vs. 78, *U* = 34.5, *p* = 0.03). In the PSQI (Table 5), the mean scores were 8.1 and 8.7 for melatonin and placebo respectively (median 8.0 vs. 9.0 respectively, *U* = 56.5, *p* = 0.89).

**Table 3:** Median and range for ICIQ NQol by treatment with p-value and standardized effect size, *d*

Measure	Treatment					
	A: Placebo		B: Melatonin		*p*	*d*
	Median	Range	Median	Range		
Overall score	25.0	8–35	20.0	8–42	0.341	0.261
Overall interference	5.0	0–9	4.0	0–9	0.444	0.209
Sleep/Energy sub-score	11.0	3–19	10.0	3–18	0.549	0.164
Bother/Concern sub-score	9.5	4–21	9.0	3–16	0.288	0.292

**Table 4:** Median and range for MSQoL by treatment, with *p*-value and standardized effect size, *d*

Measure	Treatment					
	A: Placebo		B: Melatonin		*p*	*d*
	Median	Range	Median	Range		
Mental Overall Score	68.0	16–92	75.0	28–92	0.110	0.446
Physical Overall Score	44.0	17–89	48.5	21–90	0.023	0.651
Energy Score	30.0	0–76	36.0	0–84	0.212	0.347
Emotional Wellbeing	68.0	32–100	72.0	32–100	0.961	0.013
Physical Health	45.0	5–100	40.0	5–100	0.701	0.105
Role limitations due to physical problems	12.5	0–100	25.0	0–100	0.015	0.701
Role limitations due to emotional problems	100	0–100	100	0–100	0.580	0.151
Health Perceptions	42.5	5–85	45.0	0–80	0.396	0.233
Social Function	67.0	25–100	67.0	0–100	0.603	0.142
Cognitive Function	67.0	0–100	73.0	0–100	0.884	0.040
Health Distress	60.0	15–90	60.0	0–100	0.862	0.036
Change in Health	25.0	0–50	50.0	25–100	0.647	0.125
Quality of Life	55.0	27–95	63.0	0–90	0.130	0.421
Pain Score	78.0	0–100	70.0	0–100	0.029	0.622

**Table 5:** Median and range for PSQI by treatment, with *p*-value and standardized effect size, *d*

Measure	Treatment					
	A: Placebo		B: Melatonin		*p*	*D*
	Median	Range	Median	Range		
Overall Score	9.0	2–13	8.0	2–16	0.893	0.036
Sleep Disturbances	2.0	1–3	2.0	1–3	0.092	0.471
Sleep Medication	0.0	0–0	0.0	0–3	0.228	0.333
Sleep Duration	1.0	0–3	1.0	0–3	0.663	0.119
Sleep Latency	1.0	0–5	1.0	0–3	0.927	0.025
Sleep Quality	1.0	0–3	1.0	0–3	0.922	0.027
Daytime Dysfunction	1.0	0–3	1.0	1–3	0.141	0.410
Habitual Sleep Efficiency	2.0	0–3	1.0	0–3	0.114	0.440

In all analyses there was little evidence of any carryover effects (i.e. no evidence of period by treatment interaction effects, consistent with a sufficiently large washout period). In addition, the given conclusions for treatment effects are replicated if differences are examined as paired differences using the non-parametric Wilcoxon signed rank test.

### Safety

EDSS data is given in Table 6. Overall, there was no difference in EDSS while taking melatonin or placebo; mean score was 4.4 for placebo and 4.7 for melatonin (medians 4.0 and 4.0). Four patients reported worsening symptoms of MS during the study, of which two were taking melatonin at the time. One experienced two separate episodes, once whilst taking melatonin and once whilst taking placebo. One patient experienced Uhthoff’s phenomenon, a worsening of neurological condition related to MS, while taking melatonin.

**Table 6:** Median and range for EDSS by treatment with *p*-value and standardized effect size, *d*

Measure	Treatment					
	A: Placebo		B: Melatonin		*p*	*d*
	Median	Range	Median	Range		
Visual	1.0	0–4	1.5	0–4	0.056	0.539
Brainstem	1.0	0–2	1.0	0–2	0.288	0.292
Sensory	3.0	0–4	3.0	0–3	0.090	0.474
Pyramidal	2.0	0–3	2.0	0–3	0.386	0.238
Cerebellar	2.0	1–4	2.0	0–3	0.181	0.370
Cerebral	2.0	0–3	2.0	0–3	0.653	0.123
Bladder and Bowel	2.0	1–3	2.0	0–3	0.674	0.115
Ambulatory	1.0	0–9	1.0	0–9	1.00	0.000
EDSS	4.0	1.5–8	4.0	3–8	0.209	0.347

Adverse event reporting most commonly identified urinary tract infection (UTI), which affected seven participants. Four of these were prior to receipt of study medication. Three UTIs were found after randomisation. One was on treatment phase 1 and was receiving melatonin. Another had completed treatment phase 2 (placebo). One participant was found to have a UTI after the post drug wash out phase for melatonin.

Two patients experienced faecal urgency; both were no longer taking study medication at the time of onset, and both had been taking placebo.

Two participants experienced lassitude and anergia. One was withdrawn by the clinician, and was found to have been taking placebo. The other had already completed the study, and had been taking melatonin as the most recent study medication.

One patient experienced severe dizziness, and was unblinded and was found to have been taking placebo; this patient was withdrawn from the study. A further patient reported abdominal pain (a reported potential side effect of Melatonin), and was withdrawn by the clinician without unblinding; subsequently the patient was found to have been taking placebo.

Two patients reported chest infections, one taking placebo and the other melatonin. One of these patients went on to report a further three adverse events of pain in fingers, knees and shoulder, which all occurred whilst taking placebo.

One patient experienced abdominal pain resulting in emergency department review, where cholecystitis was diagnosed. She also reported shingles and reduced mobility in separate adverse events. She had been taking the placebo on all of these occasions. One patient reported a probable olecranon bursitis while taking placebo. Colds and an ear infection were reported by two patients; both were taking placebo.

## Discussion

Nocturia is a prominent symptom with substantial detrimental impact on quality of life in MS, especially in light of the range of factors affecting sleep quality in these patients. Ordinarily, there should be a reduction in urine production rate during sleep. Endogenous melatonin is a key contributor in circadian control, and disruption of melatonin is a feature in sleep disturbance in MS [30]. We surmised that beneficial effects may result indirectly by improved sleep quality, and perhaps directly by some restoration of the normal circadian reduction in urine production at night, and reduced bladder smooth muscle activity. A potential impact of giving supplementary melatonin orally as regulator of circadian rhythms in restoring some measure of circadian control [31] could be beneficial in the proposed context. In reality, the effect of melatonin in the current study did not identify any reduction in nocturia (either objectively or subjectively). The bladder diary was the main outcome assessment, and during the active treatment phase there was no reduction in nocturia episodes or overall nocturnal urine production.

For the PSQI, the mean scores at the end of the treatment phase were 8.1 for melatonin and 8.7 for placebo. This difference was statistically significant, and seems to indicate worse function with melatonin, but the difference is modest and unlikely to be clinically significant. We also evaluated other aspects of the patient’s health and QoL. For the MSQoL, there was no significant change between melatonin and placebo in most of the domains, except that small statistically significant differences were evident in the physical overall score, role limitations due to physical problems, and pain score. It is unclear that this was a definite consequence of melatonin action.

Symptom scores were a key secondary measure, and again melatonin did not appear to have any effect on nocturia specifically or LUTS in general. Nocturia-specific quality of life did not show any evident improvement. Nocturia has a multifactorial pathophysiology, which potentially could mean that melatonin might have effect in some individuals and not others, and this was considered to explain the finding of a responder group in a previous study which examined melatonin use for treatment of nocturia in men with benign prostate enlargement [15]. However, there did not seem to be any evident responder group in the current study.

The lack of evident difference between melatonin and placebo may reflect the pragmatic approach taken in the study. Diabetes mellitus and diabetes insipidus were excluded, but otherwise inclusive criteria were used for study recruitment. This pragmatic approach was taken to reflect the utility of a therapy which could be applied to the majority of patients in the general healthcare context, without having to undertake too much clinical assessment. The fact that we did not identify benefit could reflect the wide range of potential co-morbidity in MS, indicating that it is likely that assessment of specific underlying mechanisms probably is needed to understand nocturia in MS. A low dose of melatonin was chosen (2 mg), because at the time the study was designed there was discussion that melatonin could potentially contribute to deterioration in MS severity through effects on immune function [32]. Based on the results, it seems that this low dose was insufficient for nocturia therapy, even though it is a standard dose for treating sleep disorders in a wider population. Some studies have used higher doses of melatonin in an MS population, demonstrating improved sleep quality [19]. Recent literature does not identify detrimental effect of melatonin on MS severity, and alternative hypotheses have been promulgated regarding a potential beneficial effect. We did not measure endogenous melatonin production, or effective serum melatonin levels on treated patients, so we are unable to state whether serum levels of the hormone reached therapeutic levels in our study population.

The inclusion of a placebo group is an essential part of LUTS investigation, since placebo responses are noted to be rather big generally [33]. In the current study, the placebo response seen in the bladder diaries and symptom scores was modest. Undertaking observations such as bladder diaries is considered a potential factor that could influence patient behaviour, since completing a bladder diary shows to a patient when they are generating a high urine output, which can feed back on their behaviour. This was not a particular observation in the current study, where the baseline 24 h voided volume was 2.2 L, and was similar during the treatment phases (2.2 L for placebo, and 2.0 L per 24 h for melatonin, medians 2.1 L, 2.2 L, and 2.0 L respectively). The nocturnal polyuria index was unchanged (median 0.32, 0.33, 0.32 for baseline, placebo and melatonin respectively). A small reduction in median nocturnal urine output was seen (baseline 741 mL, placebo 651 mL, and melatonin 667 mL), but this was not statistically significant. It did not yield a significant change in nocturia episodes (median 1.70, 1.50 and 1.30 respectively).

There was no clear adverse safety signal. No adverse events appeared to have any clear link to the melatonin therapy on a consistent basis. UTIs were reported, and two episodes of faecal urgency. Single presentations with Uhthoff’s phenomenon, feeling drained, cold hands, profound somnolence, and others were also described. The qualitative interviews also identified that fatigue was a key feature. The individual with Uhthoff’s phenomenon explains the difference in the visual domain on the EDSS scores, which were otherwise not significantly different for the melatonin and placebo phases.

## Conclusions

In summary, a low dose of melatonin taken at bedtime may be an ineffective therapy for nocturia in MS, studying an adult population with nocturia once per night or more often. A different dose regime of melatonin or recruitment selection criteria would need to considered to ascertain whether melatonin could influence nocturia in MS.

## Abbreviations

CaMBS: Cambridge Multiple Sclerosis Basic Score
EDSS: Expanded Disability Status Scale
FVC: Frequency volume chart
ICIQ: International Consultation on Incontinence Questionnaires
ICIQ-FLUTS: Female- specific tool for assessing severity and bother of all LUTS
ICIQ-MLUTS: Male-specific tool for assessing severity and bother of all LUTS
ICIQ-N: International Consultation on Incontinence-Nocturia
ICIQ-NQoL: A nocturia-specific quality of life tool which will be used throughout the study
LUTS: Lower urinary tract symptoms
MeNiMS: Study Acronym: Melatonin for Nocturia in Multiple Sclerosis
MS: Multiple sclerosis
MSQLI: Multiple sclerosis quality of life index
PSQI: Pittsburgh Sleep Quality Index
